# Utilization and its factors of post abortion modern contraceptive in Ethiopia: a systematic review and meta-analysis

**DOI:** 10.1186/s12978-021-01195-8

**Published:** 2021-07-03

**Authors:** Fentahun Yenealem Beyene, Azimeraw Arega Tesfu, Kihinetu Gelaye Wudineh, Fikadu Waltenigus Sendeku, Asteray Assemie Ayenew

**Affiliations:** Department of Midwifery, College of Medicine and Health Sciences, Bahir Dar University, P.O. Box 79, Bahir Dar, Ethiopia

**Keywords:** Post abortion, Utilization, Modern contraception, Systematic review, Meta-analysis, Ethiopia

## Abstract

**Background:**

Providing post-abortion care service is a widely accepted to reduce maternal morbidity and mortality by promoting, preventing and treating maternal and neonatal health, identifying the utilization and its factor of post abortion contraceptive is crucial. Therefore we tried to review post abortion contraceptive utilization and its factors in Ethiopia.

**Methods:**

A review was performed by using the Preferred Reporting Items for Systematic Reviews and Meta-Analyses (PRISMA). A systematic and a comprehensive literature searching mechanism were used without any restriction, through Google scholar, PubMed, EMBASE, Scopus, Web of Sciences, and Grey literature databases for reporting utilization of post abortion family planning. Pilo-tested were performed in random sample studies and a standardized data extraction form was used. All statistical analyses were done using STATA version 14 software for windows, and meta-analysis was used with a random-effects method. The results are presented using texts, tables and forest plots with measures of effect and 95% confidence interval.

**Results:**

Among 1221 records, 11 studies were taken in the meta-analysis with 4336 Participants that full fill the inclusion criteria. The pooled prevalence of post abortion contraceptive in Ethiopia was 74.56% (95% CI (73.31%, 75.81%)). Married women (OR 2.01 (95% CI (1.52, 2.66), I2: 0.0%)), women who were counseled (OR 5.36 (95% CI (3.10, 9.29), I2: 79.5%)), women whose educational level tertiary and above (OR 2.28 (95% CI (1.66, 3.17), I2: 0.0%)), women who had ever used contraceptive (OR 3.76 (95% CI (2.19, 6.47), I2: 67.8%)) and those women’s age 15–24 years old (OR 8.35 (95% CI (2.74, 14.74), I2: 87.4)) were statistically significant.

**Conclusion:**

According to World Health Organization (WHO) guideline, "after a miscarriage or induced abortion, the recommended minimum interval to next pregnancy is at least 6 months in order to reduce risks of adverse maternal and perinatal outcomes".. According to this post abortion contraceptive utilization in Ethiopia is not optimal. Marital status, education, Counsel, previously exposed and age were significantly associated. Therefore, the Ministry of Health should work target fully to address those problems to maintain maternal and child health in Ethiopia.

**Supplementary Information:**

The online version contains supplementary material available at 10.1186/s12978-021-01195-8.

## Plain language summary

Post abortion family planning utilization is a woman utilizes at least one of the modern contraceptive methods within 6 weeks of post abortion. Providing post-abortion care service is a widely accepted strategy to reduce maternal morbidity and mortality. Linkage between abortion care and family planning can help in preventing unwanted pregnancies and thus induced abortion may be prevented. Post abortion women are a clear need for family planning. Even if a woman wants to have a child immediately, WHO guidelines recommend; "after a miscarriage or induced abortion, the recommended minimum interval to next pregnancy is at least 6 months in order to reduce risks of adverse maternal and perinatal outcomes".. Since the main strategy of providing post abortion family planning is for promoting, preventing and treating maternal and neonatal health, identifying the utilization and its factor of post abortion contraceptive is crucial. Therefore we tried to review post abortion contraceptive utilization and its factors in Ethiopia. We reviewed 1221 records and 11 studies were fulfill the inclusion criteria and taken in the meta-analysis with 4336 participants. The pooled prevalence of post abortion contraceptive in Ethiopia was 74.56% (95% CI (73.31%, 75.81%)). Marital status, education, Counsel, previously exposed and age were significantly associated factors. As per the finding investing on women education and counseling is needed.

**Supplementary Information:**

The online version contains supplementary material available at 10.1186/s12978-021-01195-8.

## Introduction

While the goal of the Millennium Development Goal (MDG) 5 to reduce maternal mortality was 5.5%, the maternal mortality ratio trend from 1990 to 2015 showed a decrease of just 2.3%, 55% lower than expected [1]. Knowing this, maternal and child health has gained strong global attention in the field of the Sustainable Development Goal (SDGs) and the National Health Sector Transformation Plan (HSTP) [2, 3].

Globally; about 73% of maternal deaths occurred due to direct cause and 27.5% of death occurred due to indirect causes. Of such hemorrhage accounts 27.1%, hypertensive disease 14%, sepsis 10.7%, abortion 7.9% and embolism and other direct causes 12.8% [4–6].

Sub-Sahara Africa region is one of the region having high maternal and newborn death. It accounts more than half of the global maternal and newborn death [6, 7]. The MMR in low income countries in 2017 is 462 per 100,000 live births versus 11 per 100,000 live births in high income countries [8].

Ethiopia is one of the sub-Sahara Africa countries having high maternal and newborn mortality. In the country 412 maternal death per 100,000 live births were occurred annually [9]. In the world around 20% pregnancies end up with induced abortion [10]. Currently around 44 million abortion are induced annually worldwide, from this around 38 million induced abortions were in developing countries [10].

More than 40% of the total deaths due to unsafe abortion have occurred in Africa making it the leading cause of maternal mortality in the area. Abortion prevalence is higher where the unmet need for family planning is high, contraceptive prevalence is low, and less-effective contraceptive methods prevail [11]. Nearly, 20% of post abortion clients didn’t get family planning even if they need [12]. Providing post-abortion care service is a widely accepted strategy to reduce maternal morbidity and mortality. Linkage between abortion care and family planning can help in preventing unwanted pregnancies and thus induced abortion may be prevented [13].

Post abortion women are a clear need for family planning. Even if a woman wants to have a child immediately, WHO guidelines recommends; "after a miscarriage or induced abortion, the recommended minimum interval to next pregnancy is at least 6 months in order to reduce risks of adverse maternal and perinatal outcomes" [14].

The technical advisory group of international experts identified, post abortion family planning is one of several high-impact practices in family planning (HIPs) [15].

Since the main strategy of providing post abortion family planning is for promoting, preventing and treating maternal and neonatal health, identifying the utilization and its factor of post abortion contraceptive is crucial. Therefore we tried review post abortion contraceptive utilization and its factors in Ethiopia.

## Methods

### Design and search strategy

This study was carried out using a pre-specified procedure in order to analyze the evidence showing the use of post-abortion family planning and its factor in Ethiopia. To review and reporting the results of this systematic review and meta-analysis we used the Preferred Reporting Items for Systematic Reviews and Meta-Analyses (PRISMA) guidelines [16] for detail (Additional file 1).

A systematic and a comprehensive literature searching mechanism were used without any restriction from the staring of study till sending to the journal; via Google scholar, PubMed, EMBASE, Scopus, Web of Sciences, and Grey literature databases for reporting utilization of post abortion family planning and its factor. We also performed hand searched for cross-references to distinguish additional relevant articles. Our search strategy has included; “utilization of post abortion family planning”, “post abortion family planning”, “associated factors of post abortion family planning”, “post abortion care”, “unintended pregnancy”, and “Ethiopia”.

### Inclusion and exclusion criteria

#### Inclusion criteria

All published and repository studies that addressed the utilization and its factors of post abortion family planning and reported appropriate measures of association were included.

#### Exclusion criteria

Reviews, case reports, conference abstract, and letters; studies in which appropriate measures of association were not reported; abstracts with no more information or no full-text article; and duplicate data were excluded.

#### Data collection and synthesis

Two independent authors (FYB and AAT) screened eligibility of all retrieved articles through the search strategy by title and abstract based on the inclusion and exclusion criteria. In addition, the three authors (KGW, FWS and AAA) performed the quality assessment of studies. In the case of disagreement, they resolved it by logical consensus.

### Data extraction

Pilo-tested were performed in random sample studies and a standardized data extraction form was used. Data were collected on first author's, year of publication, year of data collection, study setting, study design, sample size, outcome definition, response rate, statistically significant factors, adjusted Odds ratio(AOR), 95% confidence interval, prevalence and covariance is considered. In the case of results published several times; the data were considered only once. Uncertainties during the extraction process were resolved by logical consensus between the two authors. In the case of incomplete data, we excluded the study or the parameter that was not available.

### Study quality and risk of bias assessment

The quality and strength of each study was assessed by using the development of a critical appraisal tool for use in systematic reviews addressing questions of prevalence and incidence [17] in detail (Additional file 2). We assessed based on the following assessment criteria: inclusion/exclusion criteria, sampling strategy, and sample size, sample representativeness, methods of data collection and adequacy of response rate (Table 1).

**Table 1:** Characteristics of studies which are included in the systematic review and meta-analysis, 2019

Author	Study year	Publication year	Region	Study area	Study design	Sample size	Proportion of post abortion FP
Mekuria et al	2018	2019	Amhara	Bahr Dar Town	Cross-sectional	400	78.5
Matiyas Asrat	2017	2018	Addis Ababa	Addis Ababa	Cross-sectional	552	90.6
Hagos et al	2017	2018	Tigray	Central zone towns of Tigra	Cross-sectional	409	70.9
AyeleMamo Abebe et a	2018	2019	Amhara	Dessie	Cross-sectional	118	84.1
Dejenie Seyoum et al	2013	2014	Amhara	Gondar Town	Cross-sectional	630	73.5
Abebe Muche et al	2018	2019	Amhara	Debre Birhan Town	Cross-sectional	371	45.8
Mahlet Kassahun	2017	2017	Addis Ababa	Addis Ababa	Cross-sectional	459	68.8
Moges et al	2017	2018	Tigray	Shire town	Cross-sectional	400	61.5
Erko EK et al	2015	2016	Oromia	Jimma town	Cross-sectional	184	70.1
Kokeb et al	2014	2015	Amhara	Debre-Markos	Cross-sectional	414	59.2
Abdulhakim Abamecha et al	2015	2016	Gambella	Gambella town	Cross-sectional	399	72.9

### Data synthesis and statistical analysis

For selected papers, a meta-analysis was carried out to provide a comparable classification of the outcome and determinants of interest. The effect size of the Meta-analysis was performed for the utilization of post abortion family planning in Ethiopia. Based on eligibility requirements, the associated factors of post abortion family planning utilization were analyzed. We had considered at least two studies were reported about one associated factors of post abortion family planning utilization in common with their measure of effect and 95% confidence interval (CI). The significant association between post abortion family planning utilization and its factors was estimated by calculating the effect size and 95% confidence interval (CI). In order to determine differences between the studies as well as within the studies, a random effects model based on the DerSimonian–Laird method was considered [18]. In addition, I2 statistics and Cochran's Q test have been used to measure heterogeneity through studies. The I2 statistics are considered to measure the percentage of the overall variance in the sample due to heterogeneity. The I2 value ranges from 0 to 100%, with I2 >  = 75% indicating high heterogeneity across the studies [19]. In Meta-analysis with funnel plot, we examined publication bias qualitatively, and Begg's test and Egger's test (p < 0.05) to consider statistical significance Statistical analysis was conducted using STATA version 14. The results are presented using texts, tables and forest plots with measures of effect and 95% confidence interval.

### Sensitivity and subgroup analysis

We consider possible sources of heterogeneity using sensitivity analysis of the selected studies. The effect of unacceptable studies was evaluated via sensitivity analyses. Subgroup analysis was also performed for prevalence by place of study, region and sample size.

### Operational definitions

Post abortion family planning utilization: A woman utilizes at least one of the modern contraceptive methods within 6 weeks of post abortion [14].

A modern contraceptive method was defined as using one or more of the following method(s): male and female condoms, OCPs, injectable, implants, IUDs, sub-dermal implants, male and female sterilizations, or ECPs [20].

## Results

### Description of the studies

**Fig. 1:**
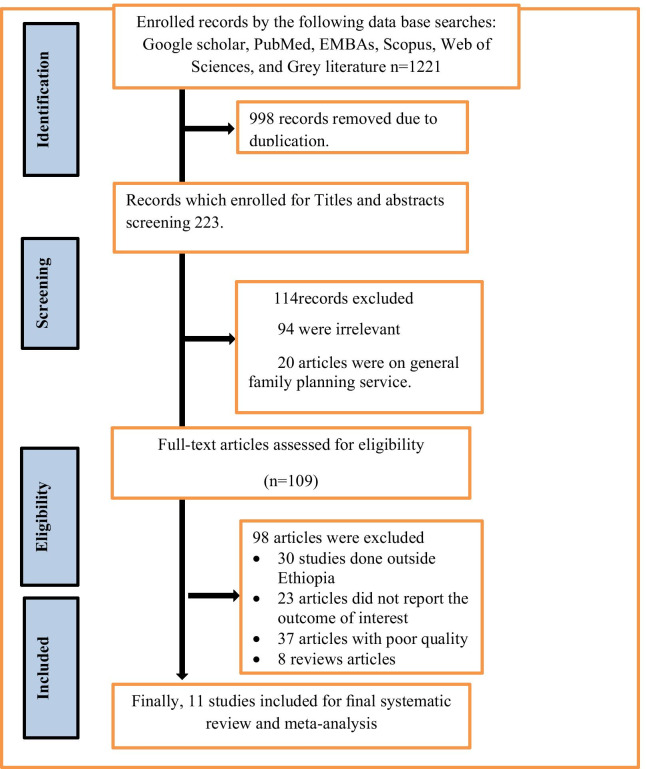
PIRSMA Flowchart diagram of the study selection

Through the use of medical electronic databases and other relevant sources, we tried to search for around 1221 studies. Among the identified studies, 998 articles were removed due to duplication, while 223 articles were considered for further screening. After curiously screening of 223 studies for their eligibility based on the title and abstract, 114 studies were excluded due to incompatibility of the content in the title and abstract. In addition to, the full text of 109 studies were assessed for their eligibility and compatibility. In the full assessment of the text, 98 studies were excluded from the review due to duplication, in appropriate use of statically analysis, inconsistent report of result, inconsistent study outcome, or irrelevant target participants. Finally, eleven studies were considered for the pooled estimation of post abortion family planning utilization and its factor analysis (Fig. 1). Among the included studies, ten of them were published articles while one of them was repository articles. All of the included studies were cross-sectional by design and institutional setting studies were conducted in a facility setting (Table 1).

A total of 11 studies with 4336 participants were included. Among those, two studies [21, 22] were conducted in Addis Ababa, five [23–27] in Amhara region, two [28, 29] in Tigre region, and the rest two [30, 31] in other regions (Oromia and Gambella).

### Utilization of post abortion family planning

**Fig. 2:**
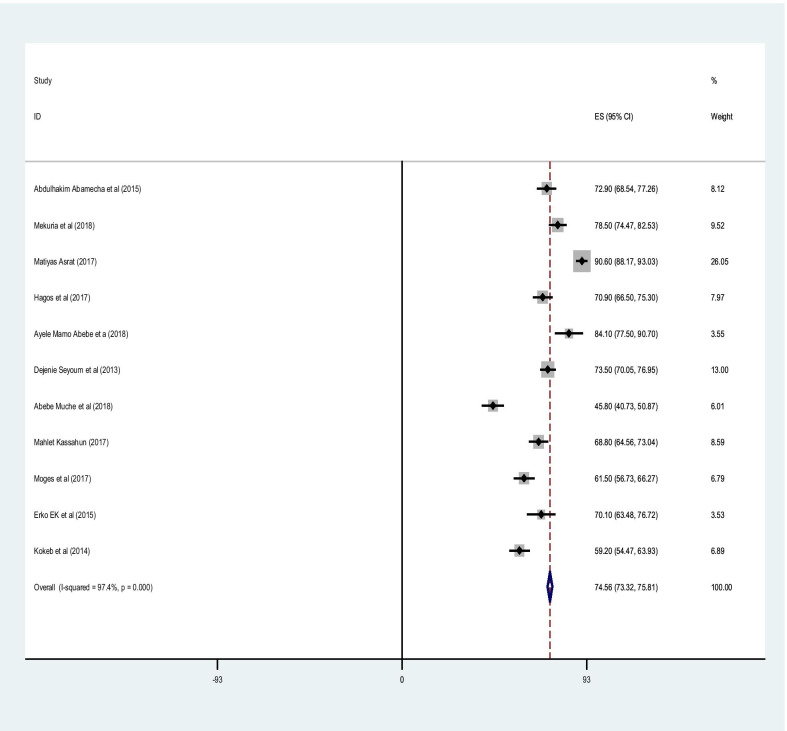
Forest plot for pooled prevalence of post abortion contraceptive

Using a random effects model, the pooled estimate of post-abortion family planning in Ethiopia was 74.56% (95% CI 73.31%, 75.81%) with significant heterogeneity between studies (I2 = 97.4, P = 0.000) (Fig. 2).

**Fig. 3:**
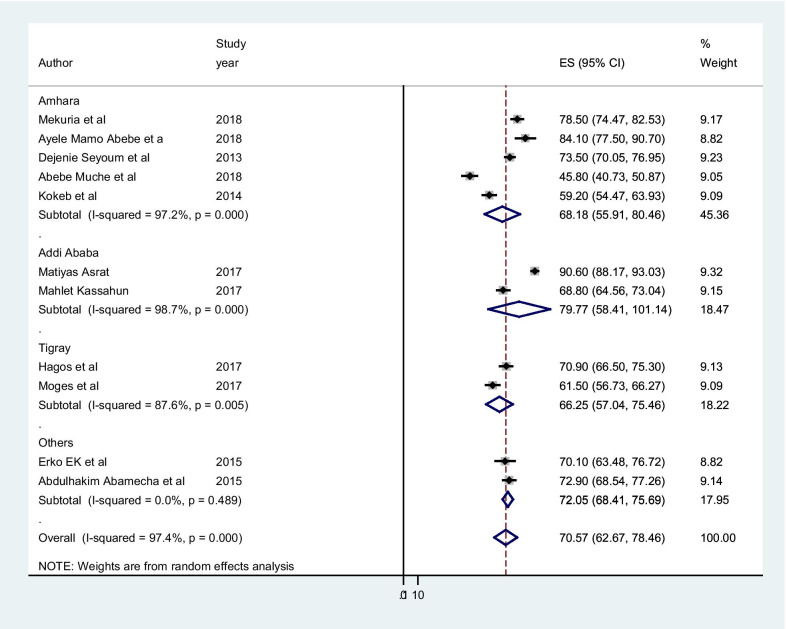
Sub group analysis by region on utilization of post abortion contraceptive

According to the subgroup analysis by region, the highest level of post abortion family planning utilization is in Addis Ababa (79.77% (95% CI 58.41, 101.14), I2 = 98.7%), while the lowest utilization was observed in Tigray region (66.25% (95% CI 57.04, 75.46), I2 = 87.6%) (Fig. 3).

### Testing heterogeneity and publication bias

**Fig. 4:**
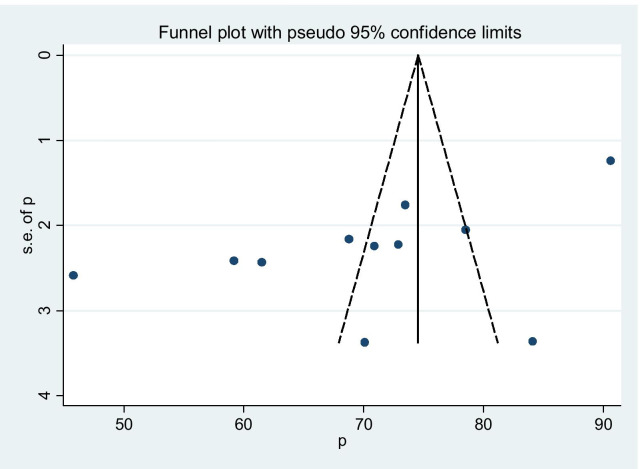
Funnel plot to show the publication bias in 11 studies

**Fig. 5:**
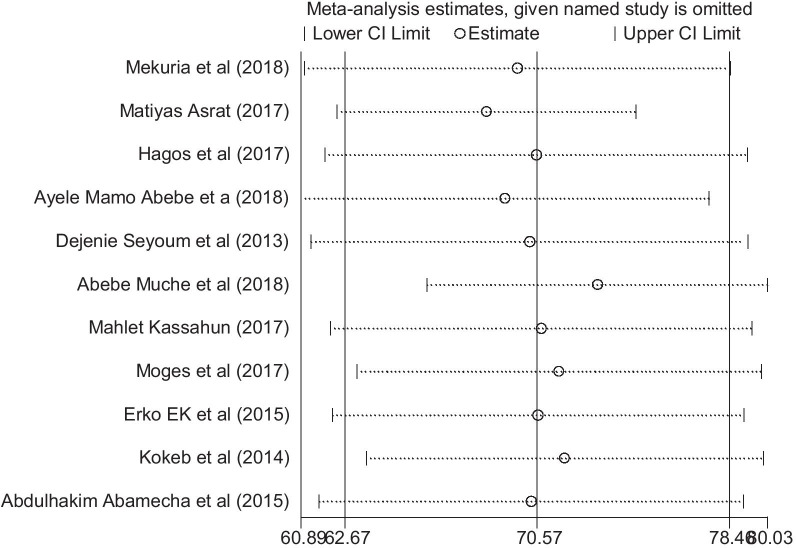
Sensitivity analysis of the 11 studies

In the overall result of this meta-analysis implies that there was statistically significant heterogeneity among studies (I2 = 97.4%), and we tried to performed a subgroup analysis by region to decrease and adjust heterogeneity (Fig. 3). The publication bias was examined using the funnel plot and Egger’s test and suggested that asymmetrical distribution of included studies (Fig. 4). However, the result of Egger’s test was statistically significant for the presence of publication bias (P = 0.0001). And also we tried to perform the sensitivity analyses using random-effects model and suggested that none of the studies influenced the overall estimate (Fig. 5).

### Factors associated with post abortion family planning utilization

**Fig. 6:**
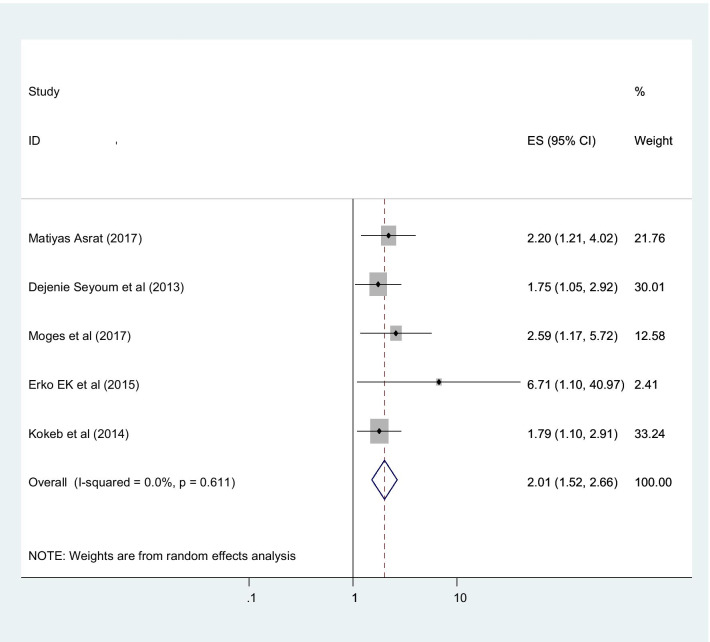
Forest plot showing the association between post abortion contraceptive utilization and married women

**Fig. 7:**
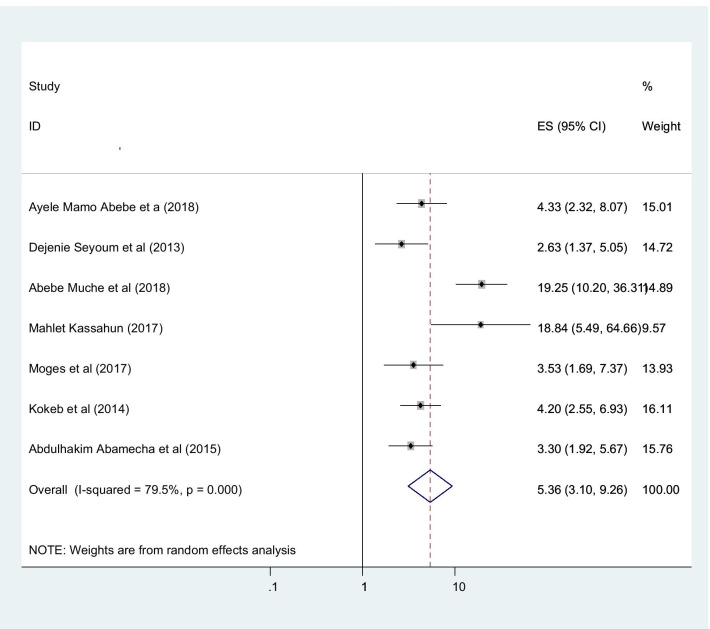
Showing that the association of post abortion contraception and Counseled women

**Fig. 8:**
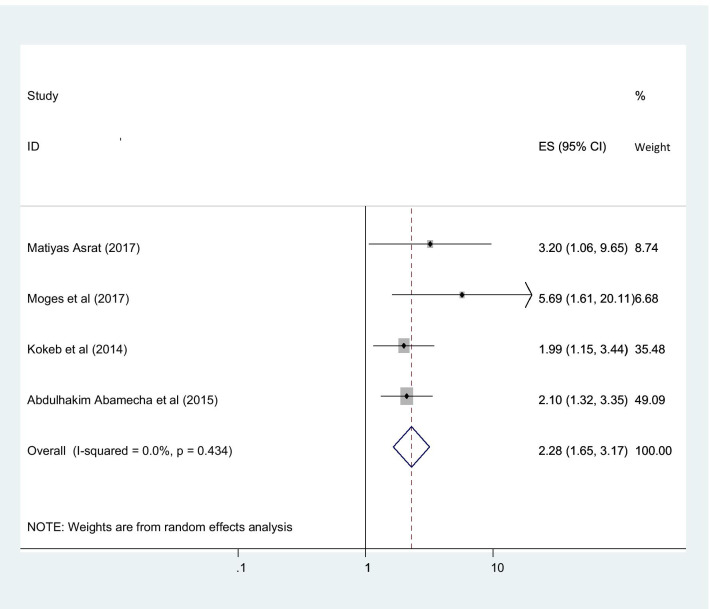
Showing the association between post abortion contraception and education

This study found that the utilization of post-abortion family planning in Ethiopia was associated with various important factors. Married women were more interested in post-abortion family planning than the counter parts in which they are single (OR 2.01 (95% CI (1.52, 2.66), I2: 0.0%)) (Fig. 6). The heterogeneity test (P = 0.486) showed that no evidence of variation across studies. The result of Egger’s test showed statistically significant publication bias (P = 0.005). Women who were counseled about post abortion family planning had higher chance of utilize post abortion family planning with compare those not counseled about post abortion family planning with statistically significant (OR 5.36 (95% CI (3.10, 9.29), I2: 79.5%)) (Fig. 7). The heterogeneity test (P = 0.731) showed that there is no significant variation across studies. The result of Egger’s test showed that no statically significant evidence of publication bias (P = 0.376). Participants who had tertiary and above educational level had higher chance of using post abortion contraceptive with compared with those women’s bellow tertiary educational level with statistically significant (OR 2.28 (95% CI (1.66,3.17), I2: 0.0%)) (Fig. 8). The heterogeneity test (P = 0.398) showed there is no a significant variation across studies. The result of Egger’s test showed no statistically significant evidence of publication bias (P = 0.071).

**Fig. 9:**
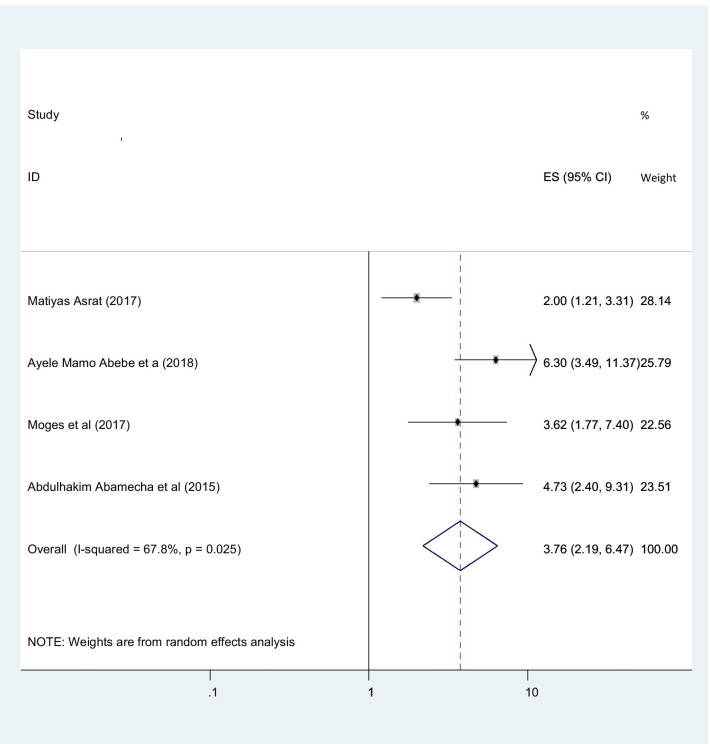
Showing the relation between post abortion contraception and ever used contraceptive

Women who had ever used contraceptive had more prevalent to participate post abortion family planning utilization than those had no any contraceptive utilization history with statistically significant (OR 3.76 (95% CI (2.19, 6.47), I2: 67.8%)) (Fig. 9). The heterogeneity test (P = 0.739) showed no a significant variation across studies. The result of Egger’s test showed no statistically significant evidence of publication bias (P = 0.404).

**Fig. 10:**
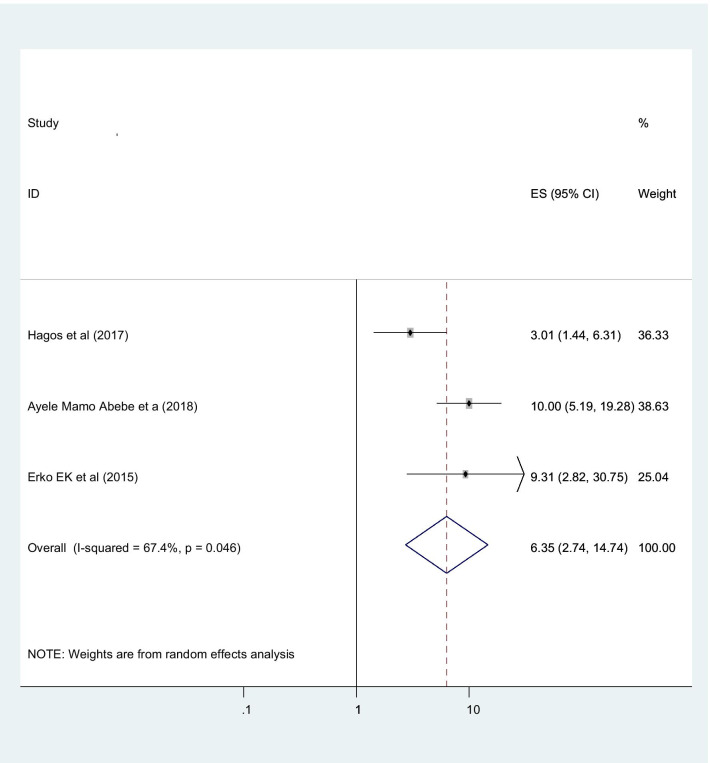
Showing that the association of post abortion contraception and age

Women whose age in between 15 and 24 years old had more utilizes post abortion contraceptive than those aged 35–44 years with statistically significant (OR 8.35 (95% CI (2.74,14.74), I2: 87.4%)) (Fig. 10). The heterogeneity test (P = 0.625) showed no variation across studies. The result of Egger’s test showed no statistically significant evidence of publication bias (P = 0.956).

## Discussion

Globally, FP has been recognized for mothers and their children as a vital lifesaving intervention and plays an important role in reducing the unmet need for FP [32].

This study found that in Ethiopia, the prevalence of post-abortion family planning was 74.56% (95% CI 73.31, 75.81). This finding was in line with studies conducted in eight African and Asian countries (73%) [33]. However, the finding of this review lower than the studies conducted in Togo (97%), Asia and sub-Saharan Africa (77%), Bangladesh averagely (91%), Brazil (80.9%), Puntland (88%), Six Indian States (81%) [34–39]; the variation might be due to variation in study population, area and methods used.

On the contrary the pooled prevalence of utilization of post abortion contraceptive higher than the studies conducted in Kumasi-Ghana (21.4%), Nepal (49.5%), Columbia (50%), Ghana (50%), and Tanzania (58%) [40–44]. This might be due to study year variation, socio-demographic characteristics of the study participants, sample size, and measurement tools used.

The subgroup analysis by region showed that the highest level of post abortion contraceptive utilization was in Addis Ababa whereas the lowest was in Tigray region. This might be due to women’s who live in the federal level were have more awareness about timing and benefit of post abortion contraceptive than those living at the regional level.

According to our review and meta-analysis finding; married women, women’s counseled about post abortion contraception, women’s who had ever used contraceptive, tertiary and above educational level and participant’s age 15–24 years old were the statically significant variables.

Married women were two times utilize post abortion contraceptive than single with OR 2.01 (95%CI (1.52, 2.66)). This finding agreement with the studies conducted in low and middle-income countries [45], Kumasi-Ghana [40]. This might be due to married women’s have a chance of discussing freely their reproductive health than single and also married women’s might get the information in multi aspects likewise from openly discussing of their parents, health professionals and colleagues.

Participants whose their education level tertiary and above were utilize post abortion contraceptive two times than bellow tertiary with OR 2.28 (95%CI (1.66–3.17)). This analysis similar with the studies in Pakistan [46], Kumasi-Ghana [40], Columbia [42], Nepal [41]. This might be the more they trained the more knowledge accessibility, the more eggers know their reproductive wellbeing, the more capability and comprehension when health professionals advise them on the advantage and timing of post-abortion contraception and having good attitudinal view of post abortion family planning than the counter parts. The possibility of receiving maternal and child health care and updating the norms and rumors is also growing as the educational level rises and health workers receive the necessary information while they are pregnant following abortion. Women counseled about post abortion contraception were five times more utilize than not counseled with OR 5.36 (95%CI (3.10–9.29)). This finding agreement with studies conducted in Puntland, Somalia [38], Columbia [42], Asia and sub-Saharan Africa [35]. This might be due to women’s who counseled might have awareness of about the appropriate timing of post abortion contraception, the drawback of early post abortion pregnancy and the benefit of post abortion contraception utilization than from non-counseled and also appropriate counseled women might have a good attitude towards post abortion contraception.

Women’s who had ever used contraceptive were more prevalent utilize post abortion contraceptive than those have no any history of contraceptive utilization with OR 3.76 (95%CI (2.19–6.47)). this agreement with the studies in Brazil [47]. This might be due to those women who were previously exposed, they have a chance using it without any influence, and they might be get appropriate information about timing of post abortion contraception than from non-users. Also those women’s who had exposure for contraceptive, they didn’t affect by rumors and might have an ability of self-determiners of their health.

Participants whose age 15–24 years old were eight times more utilize post abortion contraceptive than those age > 34 years old. This finding agrees with the studies conducted in Brazil [47]. This might be participants whose age from 15 to 24 years olds were most of them are students, daily laborer and those un ability to lead themselves; therefore these age categories participants more utilizes contraceptive until they fulfill or achieve their goal.

## Limitations

The overall finding showed significant heterogeneity, although we tried to perform subgroup analysis and meta-regression to find out the source of heterogeneity, we could not get the sources of heterogeneity. We tried to examine only the influence of five factors because other major factors were not commonly investigated by the included studies. Due to limitation of published systematic reviews and meta-analysis on utilization and its factor of post abortion contraception in national level, it creates difficulty to compare our results with other national evidence.

## Conclusions and recommendations

According to World Health Organization (WHO) guideline every woman after post abortion should stay at least for 6 months for the sack of maternal and child health. But, this systematic review showed that post abortion contraceptive utilization in Ethiopia is not optimal. Marital status, education, Counsel, previously exposed and age were significantly associated. This might be very useful for healthcare policymakers (e.g. Federal Ministry of health, Hospital administrators and NGOs) to emphasize on post abortion contraceptive utilization and to improve the maternal and child health at large. Given the multifactorial nature of factors that influencing post abortion contraceptive utilization, further qualitative research is needed to identify additional factors especially from participants perspective and explore context-specific strategies.

## Supplementary Information

**Additional file 1**: PRISMA-P (Preferred Reporting Items for Systematic review and Meta-Analysis Protocols) 2009 checklist: recommended items to address in a systematic review protocol

**Additional file 2**: Quality assessment of included studies using the Joanna Briggs Institute criteria’s for assessing quality of primary studies and JBI Critical Appraisal Checklist for Studies Reporting Prevalence Data, 2019.

## Data Availability

All the data are available from the corresponding author up on a reasonable request.

## Abbreviations

CI: Confidence interval
EOC: Emergency oral contraceptive
HIPs: High-impact practices in family planning
HSTP: Health sector transformation plan
IUD: Intra uterine device
OCP: Oral contraceptive pills
OR: Odds ratio
SDG: Sustainable development goal
PRISMA: Preferred Reporting Items for Systematic Reviews and Meta-Analyses
WHO: World Health Organization
