# Supplementary material for: Utilization and its factors of post abortion modern contraceptive in Ethiopia: a systematic review and meta-analysis
Source: Reprod Health. 2021 Jul 3;18:143. doi: 10.1186/s12978-021-01195-8 (PMC8254279; doi:10.1186/s12978-021-01195-8)
Supplement: Supplementary file 2 — Additional file 2: Quality assessment of included studies using the Joanna Briggs Institute criteria’s for assessing quality of primary studies and JBI Critical Appraisal Checklist for Studies Reporting Prevalence Data, 2019. [file 12978_2021_1195_MOESM2_ESM.docx]

Table 1: quality assessment of included studies using the Joanna Briggs Institute criteria’s for assessing quality of primary studies, 2019

| Study(Author) | Q1 | Q2 | Q3 | Q4 | Q5 | Q6 | Q7 | Q8 | Q9 | Score % |
| --- | --- | --- | --- | --- | --- | --- | --- | --- | --- | --- |
| Mekuria et al | Y | Y | Y | Y | Y | Y | N | Y | Y | 88.89 |
| Matiyas Asrat | Y | Y | Y | Y | N | Y | N | Y | Y | 77.78 |
| Hagos et al | Y | Y | Y | N | Y | Y | Y | Y | Y | 88.89 |
| Ayele Mamo Abebe et al | Y | Y | N | Y | Y | Y | Y | N | Y | 77.78 |
| Dejenie Seyoum et al | Y | Y | Y | Y | Y | Y | N | Y | Y | 88.89 |
| Abebe Muche et al | Y | Y | Y | Y | Y | N | Y | Y | Y | 88.89 |
| Mahlet Kassahun | Y | Y | Y | Y | Y | N | Y | N | Y | 77.78 |
| Moges et al | Y | Y | Y | Y | Y | Y | Y | N | Y | 88.89 |
| Erko EK et al | Y | Y | N | Y | Y | Y | Y | Y | Y | 88.89 |
| Kokeb et al | Y | Y | Y | Y | Y | Y | Y | N | Y | 77.78 |
| Abdulhakim Abamecha et al | Y | Y | Y | Y | Y | Y | N | Y | Y | 88.89 |
| Note:  Y - Yes, N - No, U – Unclear, NA- Not applicable  Q1= Was the sample frame appropriate to address the target population?  Q2= Were study participants sampled in an appropriate way?  Q3= Was the sample size adequate?  Q4= Were the study subjects and the setting described in detail?  Q5= Was the data analysis conducted with sufficient coverage of the identified sample?  Q6= Were valid methods used for the identification of the condition?  Q7= Was the condition measured in a standard, reliable way for all participants?  Q8= Was there appropriate statistical analysis?  Q9= Was the response rate adequate, and if not, was the low response rate managed appropriately? | | | | | | | | | |  |

**JBI Critical Appraisal Checklist for Studies Reporting Prevalence Data**

Reviewer Date

Author Year Record Number

|  | Yes | No | Unclear | Not applicable |
| --- | --- | --- | --- | --- |
| 1. Was the sample frame appropriate to address the target population? | □ | □ | □ | □ |
| 1. Were study participants sampled in an appropriate way? | □ | □ | □ | □ |
| 1. Was the sample size adequate? | □ | □ | □ | □ |
| 1. Were the study subjects and the setting described in detail? | □ | □ | □ | □ |
| 1. Was the data analysis conducted with sufficient coverage of the identified sample? | □ | □ | □ | □ |
| 1. Were valid methods used for the identification of the condition? | □ | □ | □ | □ |
| 1. Was the condition measured in a standard, reliable way for all participants? | □ | □ | □ | □ |
| 1. Was there appropriate statistical analysis? | □ | □ | □ | □ |
| 1. Was the response rate adequate, and if not, was the low response rate managed appropriately? | □ | □ | □ | □ |

Overall appraisal: Include □ Exclude □ Seek further info □

Comments (Including reason for exclusion)
